# Prevalence and Risk Factors for Glucose Intolerance among Saudi Women with Gestational Diabetes

**DOI:** 10.1155/2018/4282347

**Published:** 2018-08-14

**Authors:** Hayfaa Wahabi

**Affiliations:** Chair of Evidence-Based Healthcare and Knowledge Translation, College of Medicine, King Saud University, P.O. Box 2925 Riyadh 11461, Saudi Arabia

## Abstract

**Objectives:**

The objective of this study was to determine the incidence and risk factors of glucose intolerance one year after delivery in women with gestational diabetes (GDM).

**Methods:**

All women who had GDM and completed one year since delivery at King Khalid University Hospital were contacted to participate in the study. Based on to the American Diabetes Association criteria and the results of fasting blood glucose (FPG) and HbA1c, participants were classified into three groups: diabetic, impaired glucose tolerance (IGT), and normal. The incidence of diabetes and IGT was calculated. Clinical, biochemical, and sociodemographic predictors of glucose intolerance were compared between the three groups. Odds ratio (OR) for risk factors with *P* value less than 0.05 was calculated.

**Results:**

From a total 316 eligible women, 133 fulfilled the inclusion criteria and agreed to participate in the study. From the study participants, 58 (44%) women were normoglycemic, 60 (45%) women had IGT, and 15 (11%) women were diabetic. The odds of developing IGT or diabetes increased to nearly fourfold when women needed insulin for the control of GDM during pregnancy (OR 3.8, 95% CI 0.81–18.3, *P* = 0.08) and to nearly one-and-a-half-fold when they have positive family history of T2DM (OR 1.2, 95% CI 0.74–2.09, *P* = 0.40). Nevertheless, none of the odds ratios was statistically significant.

**Conclusion:**

The incidence of postpartum hyperglycemia (diabetes and IGT) is very high in Saudi women with GDM. Family history of diabetes and insulin treatment of GDM may be predictors of postpartum hyperglycemia.

## 1. Introduction

The physiological changes of pregnancy mediate carbohydrate intolerance through pregnancy-specific hormones by increasing the peripheral resistance to insulin and production of glucose; these changes call for more production of insulin to maintain normal blood glucose levels during pregnancy [1].

Gestational diabetes (GDM) is one of the commonest metabolic disorders of pregnancy. It is defined as diabetes diagnosed during pregnancy that is clearly not pregestational in nature [2].

Long follow-up of women with history of GDM showed that they are at increased risk of developing type 2 diabetes mellitus (T2DM) and cardiovascular diseases later in life [3, 4].

The majority of women with GDM convert to normal glycemic status following delivery; however, they have increased risk of developing T2DM compared to women who did not have GDM [5]. The reported rate of progression to T2DM varied considerably in different studies and is dependent on the period of follow-up, the diagnostic criteria for GDM, and the ethnicity of the studied population [6]. Risk factors for progression to T2DM include family history of T2DM, need of insulin treatment, and obesity [7–10].

It has been proven that lifestyle modification and pharmacological interventions can prevent or delay progression to T2DM in women with history of GDM [11–13]. Such interventions should be considered in the management of women with GDM especially that one in four Saudi women develops GDM during pregnancy and is among the high-risk population for developing T2DM [14].

Based on the American Diabetes Association (ADA) guidelines, different tests are equally effective for screening for impaired glucose tolerance (IGT) and diabetes including 75 oral glucose tolerance test (OGTT), fasting plasma glucose (FPG), or glycosylated hemoglobin A1C (HbA1c) [15].

There is paucity of studies on the progression to T2DM among postpartum Saudi women. The objective of this study was to determine the prevalence and risk factors of glucose intolerance one year after delivery in women with GDM.

## 2. Methods

This is a cross-sectional study conducted at King Khalid University Hospital (KKUH) in Riyadh, Saudi Arabia. KKUH is a tertiary referral hospital with 800 beds. It has all the essential departments including 20 operating theatres, an assisted reproduction unit, and a cardiac center. The hospital provides free medical care to Saudi nationals and the staff of King Saud University. The obstetrics department provides care for 3000–4000 deliveries per year.

### 2.1. Definitions

- GDM was diagnosed based on one abnormal value of 75 g on the oral glucose tolerance test (OGTT) done between 24 and 34 gestation weeks using the following World Health Organization (WHO) cutoff values [16]:
  1. Fasting plasma glucose (FPG): 5.1–6.9 mmol/l (92–125 mg/dl),
  2. 1-Hour plasma glucose ≥ 10.0 mmol/l (180 mg/dl),
  3. 2-Hour plasma glucose: 8.5–11.0 mmol/l (153–199 mg/dl).
- Diabetes and IGT were diagnosed using either fasting plasma glucose and glycosylated hemoglobin A1C (HbA1c) levels according to the following cutoff levels of the ADA [17]:
  1. Fasting plasma glucose (FPG) values:
    - Normal: <5.6 mmol/l (100 mg/dl)
    - IGT: 5.6–6.9 mmol/l (100 mg/dl to 125 mg/dl)
    - Diabetes: ≥7.0 mmol/l (126 mg/dl)
  2. HbA1c values:
    - Normal: <5.7%
    - IGT: 5.7%–6.4%
    - Diabetes: ≥6.5%
- Maternal pre-pregnancy body mass index (BMI) was calculated from maternal recall of weight prior to pregnancy and height measured during the first antenatal clinic. Current BMI was calculated using the current maternal weight and height. Participants were classified according to the following WHO BMI definitions: underweight (<18.5 kg/m^2^), normal weight (18.5–24.9 kg/m^2^), overweight (25.0–29.9 kg/m^2^), and obese (≥30 kg/m^2^) [18].

### 2.2. Participants

Each woman who delivered one year prior to the study period (September 2017–March 2018) and had GDM during the index pregnancy was invited to participate in the study.

The inclusion criteria were as follows:

1. Gestational age of 37–41 weeks at the time of delivery, calculated from the last menstrual period and/or early ultrasound scan
2. Singleton pregnancy
3. History of GDM during the index pregnancy

We excluded women with unknown glycemic status, those with pre-GDM diagnosed before or during pregnancy, women who were pregnant at the time of this study, and those who declined to participate in the study.

All women who fulfilled the inclusion criteria were contacted by phone to participate in this study. Women who agreed to participate were invited to attend a designated outpatient clinic (research clinic) fasting for at least eight hours before their appointment. The objective of the study was explained again face-to-face to each participant who subsequently signed an informed consent form. Data collected from the participants included demographic profile, obstetric history such as parity and pre-pregnancy weight, and the type of treatment of GDM in the index pregnancy (diet or insulin). Clinical data measured in each participant included current weight and height to calculate the BMI. Blood tests done for each participant were fasting blood glucose (FPG) and glycosylated hemoglobin A1c (HbA1c) levels.

Based on the results of blood tests, participants were divided into three groups: diabetic, prediabetic, and normal.

### 2.3. Statistical Analysis

Statistical analysis was performed using Statistical Package for the Social Sciences (SPSS version 22). The prevalence of diabetes and IGT was calculated. Clinical, biochemical, and sociodemographic characteristics of women who progressed to postpartum glucose intolerance (IGT and diabetes) were compared with women with normal glucose tolerance using univariate analysis. The odds ratio (OR) of the known risk factors and 95% confidence interval (CI) were estimated for factors with significant *P* value after grouping the IGT and diabetic women together in one group compared to the normoglycemic group. A *P* value of <0.05 was considered significant.

### 2.4. Ethical Approval

The study was conducted after the approval of King Saud University review board. It was conducted according to the principles expressed in the Declaration of Helsinki.

## 3. Results

From a total of 316 women with GDM who delivered one year prior to the study period, 133 met the inclusion criteria and agreed to participate in this study. From the participants, 58 women were normoglycemic, 60 had IGT, and 15 developed diabetes.

Table 1 shows the comparison of the distribution of the risk factors between the three groups. There were no significant differences between the three groups with respect to age and parity. However, there was increased frequency of risk factors when normoglycemic women were compared to those with IGT and those who developed diabetes (Table 1). The current mean BMI of normoglycemic women was significantly lower than that of the IGT and diabetic women (30.9 ± 6.4, 31.1 ± 5.6, 36.2 ± 4.8, resp., *P* < 0.01). In addition, from the women who developed postpartum diabetes, 14 (93%) had pre-pregnancy BMI of ≥30 kg/m^2^, while the corresponding proportions from the IGT group and the normoglycemic group were 24 (40%) and 31 (53%), respectively (Table 1). Significantly more women with IGT and diabetes had a family history of diabetes; in addition, they more frequently needed insulin for control of GDM during pregnancy, compared to normoglycemic women (Table 2).

Table 2 shows the odds of developing IGT or diabetes with different risk factors. The odds of developing IGT or diabetes increased to nearly fourfold when women needed insulin for the control of GDM during pregnancy and to nearly one-and-a-half-fold when they have positive family history of T2DM. On the other hand, pre-pregnancy normal BMI and diet treatment of GDM reduce the odds of developing glucose abnormalities one year postpartum. Nevertheless, none of the odds ratios for these risk factors was statistically significant (Table 2).

## 4. Discussion

The incidences of IGT and diabetes, in this study, were 45% and 11%, respectively. The results showed that women who developed glucose abnormalities (IGT and diabetes) were significantly more obese, during pregnancy and in the postpartum period. Furthermore, a significantly higher proportion from this group had a family history of T2DM and they needed insulin more frequently, for control of GDM, than the normoglycemic group.

The incidence of IGT reported in this study is higher than that reported in a national survey conducted in Saudi Arabia which showed that 25% of the adult population have prediabetes [19]. Furthermore, the incidence (new cases) of diabetes in this study is higher than the 1.3% estimated previously by Al-Quwaidhi et al. for the general population [20]. These differences may be due to the difference in the distribution of the risk factors between the general population and the participants of this study who are at higher risk of developing glucose abnormalities.

A recent study from Italy on the prevalence of glucose abnormalities in women with a history of GDM showed rates similar to those found in our study (32% and 4% for IGT and diabetes, resp.) [17]. However, a study from Iran showed lower rates than ours for both IGT and diabetes (17.5% IGT and 4.5% T2DM) [21]. These differences in rates can be explained by the variation in the criteria for diagnosing glucose intolerance, the duration of the follow-up period in addition to the ethnic differences between the studied populations [5].

Similar to previous reports, this study showed that the risk factors for developing hyperglycemia were significantly more frequent in women who developed diabetes and IGT compared to those who remained normoglycemic in the postpartum period. These include factors existing before pregnancy such as pre-pregnancy weight and a family history of T2DM, factors which occurred during the index pregnancy such as the need to use insulin to control GDM, and postpartum factors such as obesity. Although the odds for developing hyperglycemia after GDM were not statistically significant in this study, this, however, may be due to the small number of participants.

In many published reports, obesity was a significant predictor of progression to IGT and diabetes in women with GDM [16–18]. Postpartum women who are obese have 1.5-fold increase in the risk of developing diabetes in postpartum period compared to those who have normal BMI [18]. Recent published studies from Saudi Arabia showed that more than 68% of women in the reproductive age group are either overweight or obese [22] and that the prevalence of GDM and pre-GDM increases with the increase in BMI [14].

Based on a recently published systematic review, lifestyle interventions, such as healthy eating and adequate activity, are effective for the control of GDM [23]. The review showed that women who modified their lifestyle were more likely to achieve the target weight in the postnatal period and hence reduce their risk of developing hyperglycemia in the postnatal period [23, 24].

### 4.1. Strength and Limitations of the Study

This study quantified the problem of glucose intolerance following GDM in a Saudi population and provides valuable information for policymakers and researchers about the risk factors for the progression to T2DM in Saudi women. One of the limitation of this study is the relatively small number of participants compared to the total number of women who had GDM during the study period. This is due to the poor postpartum response to recall for follow-up, a problem faced by other researchers in the Middle East [25].

### 4.2. Implication to Practice

- Establishing a national program for screening women with a history of GDM for diabetes and prediabetes should be a health-service priority in a strategy for controlling the epidemic of diabetes in the country. Such programs were proven to be cost-effective considering the saving in the cost of premature death and treatment of complications of diabetes [26].
- Implementation of evidence-based national guidelines for the treatment of GDM that includes lifestyle modification is an essential step in the control of postpartum progression to T2DM.

### 4.3. Implication to Research

Research should be directed towards effective interventions to reduce the burden of obesity in Saudi pregnant and postpartum women.

### 4.4. Conclusion

The incidence of postpartum IGT and diabetes is very high in Saudi women with GDM. Treatment of GDM with insulin and a family history of T2DM may be predictors of the progression to IGT and T2DM.

## Figures and Tables

**Table 1:** Comparison of the characteristics of women with previous gestational diabetes who developed impaired glucose tolerance/diabetes and women who remained normoglycemic.

Characteristic	Normal glucose tolerance (*n* = 58)	Impaired glucose tolerance (*n* = 60)	Diabetes (*n* = 15)	*P* value
*Age (years)*				
Less than 30	18 (31)	20 (33)	2 (13)	0.41
30 or more	40 (69)	40 (67)	13 (87)	
Primiparous	15 (26)	12 (20)	2 (13)	0.71
Multiparous	43 (74)	48 (80)	13 (87)	
*Family history of DM*				
Yes	41 (71)	53 (88)	13 (87)	0.04
No	17 (29)	7 (12)	2 (13)	
*Pre-pregnancy BMI (mean ± SD)*	28.2 ± 6.4	28.4 ± 5.2	34.3 ± 5.5	<0.01
BMI ≤ 25 kg/m^2^	14 (24)	14 (23)	0 (0.0)	0.01
BMI = 25.1–29.9 kg/m^2^	13 (22)	15 (25)	1 (7)	
BMI ≥ 30 kg/m^2^	31 (53)	31 (52)	14 (93)	
Pregnancy fasting glucose level (mmol/l)	5.19 ± 0.98	5.19 ± 0.71	5.5 ± 0.7	0.35
*Treatment of GDM*				
Diet only	56 (97)	56 (93)	9 (60)	<0.01
Insulin	2 (3)	4 (7)	6 (40)	
*Current BMI (kg/m^2^)*				
BMI ≤ 25 kg/m^2^	11 (19)	7 (12)	0 (0.0)	0.22
BMI = 25.1–29.9 kg/m^2^	15 (26)	15 (25)	2 (13)	
BMI ≥ 30 kg/m^2^	32 (55)	38 (63)	13 (87)	
BMI (mean ± SD)	30.9 ± 6.4	31.4 ± 5.6	36.2 ± 4.8	<0.01

**Table 2:** Risk factors associated with the development of impaired glucose tolerance and diabetes in women with previous gestational diabetes.

Characteristic	Normoglycemic, *N* = 58 (44%)	Impaired glucose tolerance and diabetes, *N* = 75 (56%)	Odds ratio (95% CI)	*P* value
Pre-pregnancy BMI ≥ 30 kg/m^2^	31 (53%)	38 (51%)	0.94 (0.52–1.7)	0.8
Pre-pregnancy BMI < 25 kg/m^2^	14 (24%)	14 (23%)	0.78 (0.34–1.75)	0.5
Insulin treatment of GDM	2 (3%)	10 (13%)	3.8 (0.81–18.3)	0.08
Diet treatment of GDM	56 (97%)	65 (87%)	o.89 (0.54–1.47)	0.66
Family history of T2DM	41 (71%)	66 (88%)	1.2 (0.74–2.09)	0.40

## Data Availability

The data used to support the findings of this study are included within the article.
